# Effects of arbuscular mycorrhizal fungi on plant growth and herbivore infestation depend on availability of soil water and nutrients

**DOI:** 10.3389/fpls.2023.1101932

**Published:** 2023-01-26

**Authors:** Minggang Wang, Zhongbin Wang, Mingjie Guo, Laiye Qu, Arjen Biere

**Affiliations:** ^1^ Ministry of Education Key Laboratory of Silviculture and Conservation, Beijing Forestry University, Beijing, China; ^2^ Ecological Observation and Research Station of Heilongjiang Sanjiang Plain Wetlands, National Forestry and Grassland Administration, Beijing Forestry University, Beijing, China; ^3^ State Key Laboratory of Urban and Regional Ecology, Research Center for Eco-Environmental Science, Chinese Academy of Sciences, Beijing, China; ^4^ College of Resources and Environment, University of Chinese Academy of Sciences, Beijing, China; ^5^ Department of Terrestrial Ecology, Netherlands Institute of Ecology (NIOO-KNAW), Wageningen, Netherlands

**Keywords:** arbuscular mycorrhizal fungi, P fertilization, water addition, *Funneliformis mosseae*, nursery shrub species

## Abstract

**Introduction:**

Fitness of plants is affected by their symbiotic interactions with arbuscular mycorrhizal fungi (AMF), and such effects are highly dependent on the environmental context.

**Methods:**

In the current study, we inoculated the nursery shrub species *Artemisia ordosica* with AMF species *Funneliformis mosseae* under contrasting levels of soil water and nutrients (diammonium phosphate fertilization), to assess their effects on plant growth, physiology and natural infestation by herbivores.

**Results:**

Overall, plant biomass was synergistically enhanced by increasing soil water and soil nutrient levels. However, plant height was surprisingly repressed by AMF inoculation, but only under low water conditions. Similarly, plant biomass was also reduced by AMF but only under low water and nutrient conditions. Furthermore, AMF significantly reduced leaf phosphorus levels, that were strongly enhanced under high nutrient conditions, but had only minor effects on leaf chlorophyll and proline levels. Under low water and nutrient conditions, specific root length was enhanced, but average root diameter was decreased by AMF inoculation. The negative effects of AMF on plant growth at low water and nutrient levels may indicate that under these conditions AMF inoculation does not strongly contribute to nutrient and water acquisition. On the contrary, the AMF might have suppressed the direct pathway of water and nutrient absorption by the plant roots themselves despite low levels of mycorrhizal colonization. AMF inoculation reduced the abundance of the foliar herbivore *Chrysolina aeruginosa* on plants that had been grown on the low nutrient soil, but not on high nutrient soil. Fertilization enhanced the abundance of this herbivore but only in plants that had received the high water treatment. The lower abundance of the herbivore on AMF plants could be related to their decreased leaf P content. In conclusion, our results indicate that AMF negatively affect the growth of *Artemisia ordosica* but makes them less attractive to a dominant herbivore.

**Discussion:**

Our study highlights that plant responses to AMF depend not only on the environmental context, but that the direction of the responses can differ for different components of plant performance (growth vs. defense).

## Introduction

Nearly 90% of terrestrial plants are able to form a symbiosis with arbuscular mycorrhizal fungi (AMF), which is considered evolutionarily important for plants to cope with many environmental challenges (Begum et al., 2019). In exchange for providing photosynthetic carbon in the form of lipids and sugars to AMF, plants obtain water and nutrients such as phosphorus, nitrogen and micronutrients from the fungus, that can utilize its complex hyphal network to forage for these limiting resources beyond the root zone (Smith and Read, 2008). Symbiotic associations between plants and AMF have been intensively studied, and a wide range of benefits from the symbiosis in terms of plant growth have been reported. These benefits include enhancement of plant uptake of water and nutrients, as well as the promotion of tolerance to environmental stresses like drought, salinity, heavy metal contamination, shade and extreme temperature (Mathur et al., 2018; Evelin et al., 2019; Pasbani et al., 2020; Adeyemi et al., 2021; Begum et al., 2021; Saha et al., 2022). In addition to enhancing plant tolerance to abiotic challenges, AMF are also identified to play important roles in protecting plants against many types of biotic stresses, e.g. pathogen infection and herbivore feeding (Koricheva et al., 2009; Bonfante and Genre, 2010). The mitigation of effects of biotic stress by AMF operates *via* a large set of induced or primed morphological, physiological and biochemical changes in response to AMF colonization, including alterations in plant size, phenology, nutrition, palatability, digestibility and toxicity (Qu et al., 2021).

Drought is among the most frequent and devastating stresses plants experience globally (IPCC 2018), and its associated soil desiccation often causes strong plant growth depression by inducing closure of stomata and decreases in CO_2_ flux for photosynthesis (Osakabe et al., 2014; Chitarra et al., 2016). Many studies have shown that mycorrhizal plants may utilize AMF hyphal networks as extension of their root systems to scavenge water beyond the root depletion zone, allowing these plants to overcome the drought-induced depression (Ruth et al., 2011; Zou et al., 2017; Mickan et al., 2021). For example, AMF inoculation to strawberry plants under drought was shown to successfully restore plant growth to similar or higher levels compared to well-watered non-mycorrhizal plants (Boyer et al., 2015). Similar results were found in tobacco seedlings where AMF inoculation significantly decreased negative effects of drought stress and accordingly increased plant growth (Liu et al., 2021). Such AMF-mediated enhancement of plant drought tolerance can be either attributed to higher water uptake efficiency as a result of AMF extra-radical mycelia, or due to AMF-activated plant adaptation to drought in the form of multiple functional traits (Nath et al., 2016; Ruiz-Lozano et al., 2016; Liu et al., 2018; Wu et al., 2019; Zou et al., 2021).

In addition to providing drought tolerance, AMF are well known to assist the plants in the uptake of nutrients, in particular phosphorus, nitrogen and specific micronutrients (reviewed in Bucking and Kafle, 2015; Xie et al., 2022). Inorganic phosphorus (Pi) is a key nutrient that is essential for various plant functions but not easily accessible to plants due to its low solubility and mobility in soil. Many plants cannot absorb sufficient Pi for structural or metabolic use *via* their own root system (direct pathway) and partly rely on uptake of Pi through the AMF hyphal network (the mycorrhizal pathway) (Ferrol et al., 2019). Although AMF symbiosis has traditionally been considered as a mutualistic interaction, the outcome of the association for the plant can range from mutualism to antagonism, depending on environmental conditions (Smith et al., 2010). AMF-induced plant growth depressions have been reported in environments where costs of the association outweigh the benefits for plants, e.g. under low light conditions (Konvalinková́ and Jansa, 2016) and in P rich systems (Johnson et al., 2015). For instance, Schroeder and Janos (2005) showed that root colonization and growth benefit of red pepper and acorn squash from AMF was strongly reduced with increasing amounts of soil P. Similar results were found in studies of Johnson et al. (2015) who showed that mutualism between *Andropogon gerardii* and AMF only occurred in P-deficient soil. The mechanisms underlying antagonistic plant-AMF associations are still in debate (Johnson, 2010; Jin et al., 2017; Řezáčová et al., 2017; Zhao et al., 2019; Kaur et al., 2022). Traditionally, failures to observe positive mycorrhizal growth responses have been explained as cases in which the net costs of the symbiosis in terms of fungal carbon use outweigh the net benefits in terms of P delivery by the fungus to the plant *via* the AM pathway (imbalanced C-P trade). However, observations of negative mycorrhizal growth responses even when P-transfer was highly functional, have shifted this paradigm. AMF symbiosis commonly results in a repression of the plant’s own (direct) P-uptake pathway. Negative mycorrhizal growth responses are currently thought to arise from a reduction in P delivery *via* the plant’s direct pathway that is insufficiently compensated by P-uptake *via* the AM pathway (Smith et al., 2009).

Moreover, recent studies have shown that not only plant growth benefits but also plant defense benefits from AMF partners depend on environmental conditions (Bernaola and Stout, 2019; Diaz et al., 2021, Qu et al., 2021). AMF can prime plants, i.e., sensitize their immune system for stronger or faster responses to upcoming herbivores (Pozo and Azcon-Aguilar, 2007; Jung et al., 2012; Song et al., 2013), or induce higher levels of plant defensive metabolites that reduce the damage or population size of concurrent or later arriving herbivores (Vannette et al., 2013; Wang et al., 2015; Sharma et al., 2017; Meier and Hunter, 2018; Kaur and Suseela, 2020). Other studies have shown that mycorrhizal plants tend to have enhanced tolerance to herbivory (Dowarah et al., 2022) or are better at recruiting beneficial organisms (Ujvari et al., 2021). Regardless of the underlying mechanisms, the occurrence and strength of AMF effects on plant defense is often affected by the environment in which the host plants and AMF interact, leading to difficulties in application of mycorrhizal inoculation to consistently control pests in field (Delavaux et al., 2017; Bernaola and Stout, 2019). The availability of soil phosphorus and water are two major environmental factors that have individually been shown to determine the outcome of AMF-induced defense (Irankhah et al., 2020; Qu et al., 2021). However, their interactive effects on growth and defense, i.e., whether the effect of one factor depends on the presence of the other, are largely unknown.

In the current study, we manipulated soil water and nutrient content to examine how they individually and interactively affect the effects of AMF on growth and herbivore abundance of a dominant nursery shrub species *Artemisia ordosica* that dominates in semiarid region NW China. We hypothesize that (1) plants have overall enhanced growth and reduced herbivore abundance when roots are colonized by AMF, and these effects are stronger at relatively low levels of soil nutrients and water; (2) the impact of soil nutrient level on AMF benefits in terms of growth and lower herbivore abundance depends on soil water content, and *vice versa*.

## Materials and methods

### Plant, soil and AMF species

*Artemisia ordosica* Krosch. (Asteraceae) is a woody species that is widely distributed in the fixed and semi-fixed sand dunes of northwestern China. This species is a deciduous, multi-stemmed, dwarf shrub that has plumose, linearly lobate leaves and a branch system that consists of old brown branches and purple current-year twigs near the soil surface (She et al., 2017). *A. ordosica* has a deep taproot system that can reach a depth of 1–3 m, but its lateral roots are mainly distributed in the upper soil layer of 0–30 cm, and limited to a range of 0.4 m in diameter from the trunk (Zhang et al., 2008). *A. ordosica* is a typical shrub species in Mu Us desert, China and is widely used as a nursery plant species for soil restoration in this region (Li et al., 2011). The leaves of this species often start to expand in early April and the shoot biomass reaches its maximum in July, followed by a flowering season from August to late September and leaf abscission in mid-November. The species is wind dispersed by tiny light seeds over distances up to several miles. In our study, seeds of *A. ordosica* were collected from wild individuals at Shapotou Desert Experimental Station of the Chinese Academy Sciences, Ningxia Province China (104°43’8”E, 37°26’28”N) in October 2016, where the annual mean precipitation is 191 mm and the annual average temperature is 10.0°C. The collected seeds were kept dry in the dark at room temperature until use.

Soils were collected from a semi-arid restoration field near the Shapotou research station, where *A. ordosica* is the dominant species. On average, soils from this field have a water content of 1.1%, a C/N ratio of 17.0, and contain 0.28 g/kg total N and P, and 5.31 mg/kg available N and 1.95 mg/kg available P. A soil core borer of 5 cm in diameter was used to collect a total of ca. 600 kg of soil from the 0-15 cm layer. After being collected, soils were fully mixed and immediately sieved through a 5 mm mesh and sterilized in an autoclave. A thorough sterilization of soil often requires that the standard mode of autoclaving (121°C and 100 Kpa for 1 hour) is repeated two or three times. To minimize the time for sterilization yet ensuring thorough sterilization, we instead used a slightly stronger mode of autoclaving at 130°C and 200 Kpa for 15 min. Heat sterilization of soil can change soil physico-chemical properties including increases in pH, electrical conductivity, release of macronutrients, and changes in soil organic matter such as increases in the levels of humic acids. These changes may have affected plant interactions with AMF. For instance, increased release of phosphorus may reduce AMF colonization whereas higher levels of humic acids can often be well combined with successful establishment and growth promoting effects of AMF (Nobre et al., 2013; Cozzolino et al., 2016). Sterilized soils were stored at room temperature until use. *Funneliformis mosseae* was used as the source of AM fungal inoculum in this study. This species forms symbioses with a wide range of plant species, including *A. ordosica* and was identified as one of the dominant AMF species in the rhizosphere of *A. ordosica* (Qian and He, 2009). *F. mosseae*-BGC NM04A was purchased from the Institute of Plant Nutrition and Resource in Beijing Academy of Agriculture and Forestry Sciences, Beijing, China. The strain was originally collected from another semi-arid field site at Ejin Horo Banner, Inner Mongonia, China, ca. 400 km away from the experimental field, with a mean annual precipitation of 346 mm and average annual temperature of 6.3°C.

### Experimental design

In total, 128 pots were prepared and each pot was filled with 4 kg sterilized soil. The pots were assigned to eight treatments representing a full-factorial combination of two mycorrhizal treatments (M+/-), two water treatments (W+/-) and two fertilization treatments (F+/-). Each treatment had 16 biological replicates (2M × 2W × 2F × 16 replicates = 128 pots). To create the fertilization treatment (F+), half of randomly selected pots were individually fertilized with 0.0966 g granules of (NH4)_2_HPO_4_ and thus ca. 23 mg phosphate and 21 mg nitrogen was added to each pot. The other half of the pots did not receive the fertilizer and was used as the non-fertilized treatment (F-). Therefore, following the fertilization treatment there was 10.56 mg/kg available N and 7.70 mg/kg available P in F+ soils, and 5.31 mg/kg available N and 1.95 mg/kg available P in F- soils. Tap water was used to create water treatments: half of the fertilized and non-fertilized pots individually received tap water every other day, adjusting the soil water content to 4.5% (W+). The water content of the other half of the pots was adjusted to 1.5% (W-). The water contents (4.5% vs. 1.5%) were calculated from the estimated highest and lowest percentage of water retention capacity of the soil in our experimental region within a typical growing season. The amount of added water was calculated by weighing the experimental pot and estimating plant weight from a modeled plant growth curve. The mycorrhizal treatment (M+) was created by adding fifteen grams of vital *Funneliformis mosseae* inoculum contained in granules consisting of hyphae, spores and substrate to half pots of each above-mentioned water or phosphate treatment prior to seedling transplantation. The other half of the pots received sterilized inocula to create the non-mycorrhizal treatment (M-).

### Greenhouse bioassays

Seeds of *A. ordosica* were surface-sterilized using 0.1% KMnO_4_ for 30 min and rinsed twice in distilled water. Sterilized seeds were air-dried and germinated in commercial culture soils (Green Energy Inc., Wuzhong, China) at 20°C and a 16 h photoperiod for 6 weeks. Similar-sized seedlings were selected and individually transplanted into plastic pots (diameter 25cm, 16.5 cm height). All pots were grouped into 16 blocks and each block consists of one replicate from each of the eight treatment combinations. All the pots were fully watered with distilled water in the early 11 days after transplantation to ensure survival of seedlings, and dead plants were immediately replaced. After this early period when all seedlings successfully survived, plants started to receive the different watering regimes to establish the watering treatments (W+/-). Following the watering treatments, height of all plants was measured every two weeks for the next 90 days. Plants were grown in a greenhouse with a 16 h photoperiod. Natural daylight was automatically supplemented with light from 400-W metal halide lamps to keep the light intensity at ca. 2000 μmol m^−1^s^−1^ photon flux density. After these measurements, pots from block 1-8 were transplanted to the field to assess herbivore abundance. Those from the other blocks (block 9-16) were harvested to measure plant traits (see below).

### Field bioassay

The pots from block 1-8 were transferred to an undisturbed field site where *A. ordosica* populations were naturally distributed. Pots were dug into the soil with their tops leveling the soil surface and arranged in blocks at least 20 m apart according to their original block identity, with a distance of approximately 80 cm between two pots. Plants received no further fertilization or watering treatment and were naturally exposed to herbivorous insects for 4 days. After this period, plants were individually investigated to examine their colonization by herbivorous insects. Adults of the chrysomelid beetle *Chrysolina aeruginosa* Fald (Coleoptera: Chrysomelidae) showed high colonization whereas no other herbivorous insect species were found. Therefore, the numbers of *C. aeruginosa* were recorded as a measure of herbivore abundance.

### Measurements of plant functional traits

Plant shoot biomass - During the harvest of the greenhouse plants, each plant was separated into roots, stems and leaves. Plant leaves and stems were separately collected and oven dried at 70°C for 72 h to determine their dry weights. Plant roots were gently washed to remove soil particles and treated as described below.

Root architecture - Intact fresh roots of each plant were individually scanned using an Epson Perfection 4990 Photo scanner. The obtained photos were analyzed with WinRHIZO software (Regent Instruments Inc., Quebec, Canada) to estimate specific root length (SRL), root surface area (RSA), total root volume (TRV) and average root diameter (ARD). Specific root length (SRL) was calculated as total root length divided by total root biomass of an individual plant.

Root biomass - A subset of lateral roots was randomly selected from each plant. After recording their fresh weight, they were stored in 70% alcohol to determine root colonization by *F. mosseae* (see below). For the remaining roots of each plant, we measured both fresh weight and dry weight after oven drying at 70°C for 72 h. The dry-to-fresh weight ratio of these remaining roots was used to calculate the dry weight of the corresponding root subsample that was used for determining AMF colonization of the corresponding plant.

Mycorrhizal infection - Root colonization by *F. mosseae* was quantified using a gridline intersection method (Bierman and Linderman, 1981). Briefly, each stored fresh root subsample was cut into at least 100 segments of 0.5-1 cm in length. These root segments were cleared in 10% KOH for 10 min at 95°C, and stained using vinegar (5% acetic acid) and 5% black ink (Hero 440, Shanghai, China) for 8 min at 80–90°C. Stained roots were mounted on slides and checked for mycorrhizal structures (arbuscules, vesicles, spores and intercellular hyphae) under a compound microscope (BH-2; Olympus, Tokyo, Japan) at ×40 magnification. Presence of any of these structures was scored at 100 grid intersections, and mycorrhizal colonization rate of an individual plant was quantified as the percentage of intersections with mycorrhizal structures present (Wang et al., 2015).

Element analyses - Dried plant tissues, viz. leaves, stems and roots, were separately ground to a powder and 1 mg of the powder was weighed into tin capsules. The total nitrogen (N) content was measured using an elemental analyzer (Vario EL III, Elementar, Germany). Tissue samples were acid digested using a mixed solution of H_2_SO_4_ and H_2_O_2_ in a microwave oven, which continued until the samples were fully dissolved in the solution. The phosphorus (P) content in plant tissues was determined by ICP-OES (Optima 8300, PerkinElmer, USA).

Photosynthetic pigment - Leaf chlorophyll a (Chl-a), chlorophyll b (Chl-b) and carotenoid (Car) contents were determined according to the method described in Lichtenthaler and Welburn (1983) using 80% acetone as extraction solution. The Chl-a, Chl-b and Car contents were measured by spectrophotometry at wavelengths of 663, 645 and 480 nm, respectively (T60U, PG Instruments Ltd, Japan). Leaf proline content was measured using the acidninhydrine method described in Bates (1973). In brief, 0.25 g fresh leaf sample was weighed and homogenized in 5 ml 3% sulfosalicylic acid at 100°C for 10 min before filtration. L-Proline was used as a standard for the colorimetric determination of the filtrated solution at a wavelength of 520 nm.

### Statistical analyses

Plant functional traits, including plant biomass, root traits, and leaf N and P concentrations were analyzed using a general linear mixed model (GLMM) with a maximum-likelihood (ML) iterative algorithm in R version 3.6.1. In this model, AMF inoculation (M+/-), fertilization (F+/-) and water (W+/-) additions as well as their interactions were added as fixed factors, and the identity of the block to which the plants were assigned was added as a random factor. The model was run using the *lmer* function in the “lme4” package (Bates et al., 2015), and the significance of the test was estimated using the *anova* function in the “lmerTest” package (Kuznetsova et al., 2017). In the above models, data on leaf biomass and leaf and stem P content were log-transformed, and data on N content, root P content and SRL were square root-transformed prior to the analyses to meet assumptions of normality of the residual distribution and homogeneity of variances in data.

A repeated measures ANOVA (Zar, 1999) was used to analyze data on plant height, in which the treatments M, F and W were included as fixed factors and plant identity as a repeated subject. Data on insect abundance were analyzed using a generalized linear mixed model, following a “poisson” distribution, where M, F and W as well as their interactions were included as fixed factors, and block and plant height at the time of exposure to insect as random factors. A similar model was run without plant height as a random factor. The models were run using the *glm* function and the significance of the tests was estimated using the *Anova* function in the “car” package (Fox and Weisberg, 2019). All analyses were performed using R version 4.2.1 (R Core Development Team, 2022).

## Results

### AMF colonization

Overall, AMF colonization was relatively low in mycorrhizal plants (ca. 5% on average). Surprisingly, roots of plants from the non-mycorrhizal treatments were also colonized, but their colonization rate was significantly lower than that of plants from the mycorrhizal treatments (F = 4.85, p = 0.036, **Figure S1**). Root colonization by AMF was not affected by fertilization and water supply, nor by their interaction (all p >0.20).

### Plant growth

**Figure 1:**
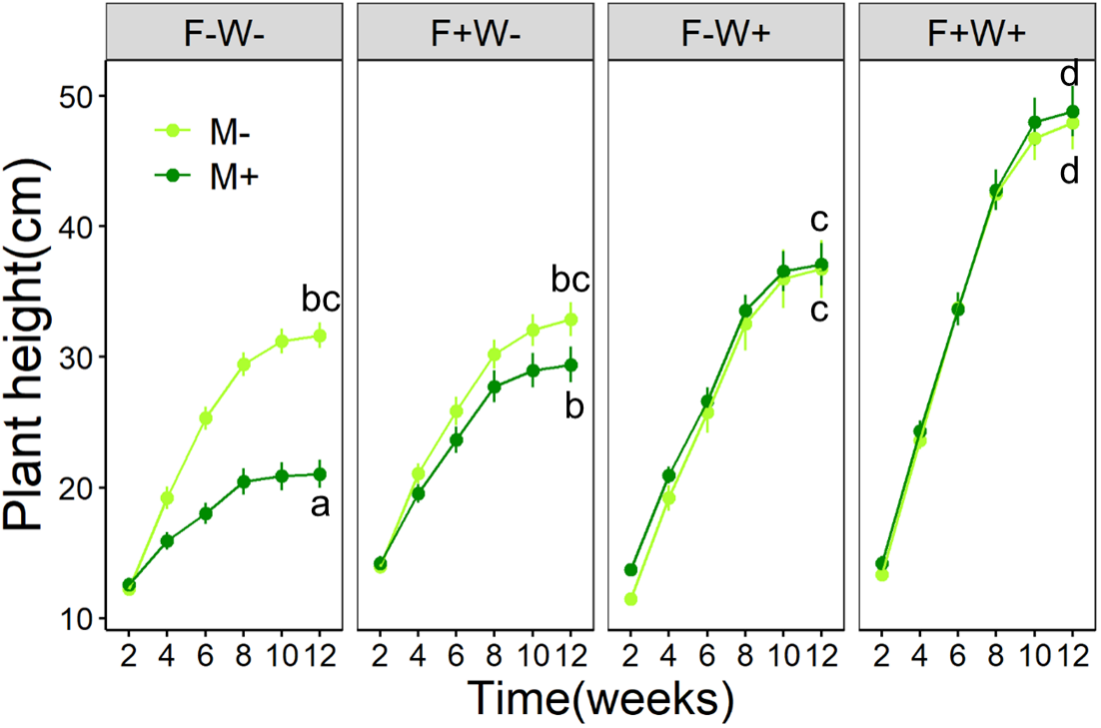
Development of plant height over time of *Artemisia ordosica* plants that were inoculated with the AMF species *Funneliformis mosseae* (M+) or not (M-) and that were grown under four different combinations of fertilization (F-: no fertilization; F+: fertilization) and water addition (W-: low soil water; W+: high soil water) treatments in a greenhouse. Light symbols: plants without AMF; dark symbols: plants with AMF. Data at the last time point (Week 12) were separately analyzed across all treatments in plot facets and the scatterplot with identical letters are not significantly different based on Tukey’s *post hoc* test. Statistics are shown in **Table 1**.

Plant height – Plant height at the harvest was significantly enhanced by both additional water supply (+10.0%) and by additional fertilizer supply (+4.5%), and their effects acted synergistically (+20.8%) (**Table 1**, **Figure 1**, W × P interaction, p < 0.01). Surprisingly, plant height was unaffected by AMF inoculation when plants received additional water, but was reduced by AMF when additional water was not supplied (**Table 1**, **Figure 1**, M × W interaction, p < 0.001). The reduction in plant height by AMF under low water conditions tended to be stronger in plants that also did not receive fertilization (-33.5%) than in plants that received the fertilizer treatment (-10.6%).

**Table 1:** Effects of fertilization (F), water addition (W) and inoculation with arbuscular mycorrhizal fungi (M) on plant height (repeated measures ANOVA) and on plant biomass, root traits and photosynthetic traits (General Linear Mixed Models, GLMM) of *Artemisia ordosica* plants grown in the greenhouse.

Source	*df*	Plant growth traits					Photosynthesis traits				Root traits			
		Height	Leaf	Stem	Root	Total	Chl-a	Chl-b	Car	Proline	SRL	ARD	RSA	TRV
**M**	1	5.60^*^	15.0^***^	10.0^**^	1.11	4.48^*^	0.06	0.12	0.05	3.38	4.47^*^	2.72	0.05	0.43
**F**	1	54.4^***^	146.3^***^	119.0^***^	14.0^**^	60.0^***^	0.26	0.88	0.00	3.36	60.0^***^	21.2^***^	0.86	2.94
**W**	1	117.1^***^	157.8^***^	237.0^***^	94.6^***^	162.7^***^	2.08	15.8^***^	8.84^**^	8.84^**^	162.7^***^	40.3^***^	40.2^***^	53.1^***^
**M×F**	1	1.95	3.95	8.79^**^	2.03	4.56^*^	0.57	2.34	1.50	0.86	4.56^*^	6.63^*^	0.02	0.41
**M×W**	1	12.7^***^	6.67^*^	14.6^***^	1.03	2.68	2.07	0.06	1.15	3.39	2.68	10.2^**^	0.13	1.03
**F×W**	1	7.26^**^	6.07^*^	32.3^***^	16.6^***^	22.0^***^	0.62	4.47^*^	3.12	0.60	22.0^***^	30.8^***^	8.23^**^	14.0^**^
**M×F×W**	1	2.94^+^	0.00	1.12	0.36	0.36	0.09	0.00	0.25	6.23^*^	0.36	0.63	0.00	0.07

F-values are shown in the table and those in bold indicate significant treatment effects (P < 0.05). ^+^p < 0.10, ^*^P < 0.05, ^**^P < 0.01, ^***^P < 0.001; df, numerator degrees of freedom; Chl-a, chlorophyll a; Chl-b, chlorophyll b; Car, carotenoid; TRV, Total root volume; RSA, root surface area; SRL, specific root length; ARD, average root diameter. There were 16 replicates in each of AMF, P, and W treatments for plant height trait, 8 replicates for plant biomass traits, and 4 replicates for root and photosynthetic traits. Plant height was repeatedly measured over time; effects of time and its interactions with P, W and AMF are not presented in the table.

**Figure 2:**
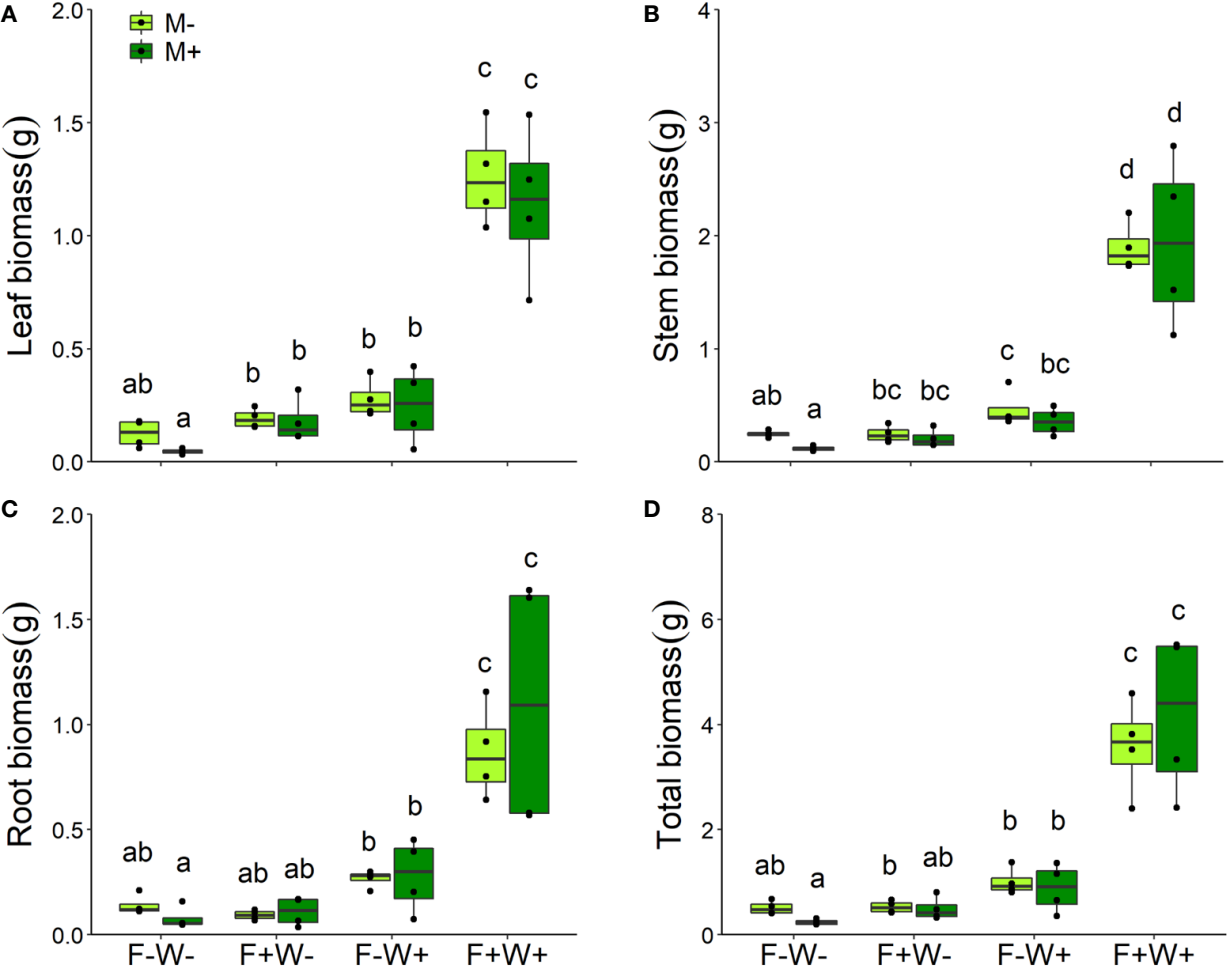
Leaf **(A)**, stem **(B)**, root **(C)** biomass and total mass **(D)** of *Artemisia ordosica* plants inoculated with AMF species *Funneliformis mosseae* (M+) or not (M-) and grown under four different combinations of fertilization (F-: no fertilization; F+: fertilization) and water addition (W-: low soil water; W+: high soil water) treatments. Light symbols: plants received no AMF; dark symbols: plants inoculated with AMF. The boxplot shows the 25% and 75% quartiles, the median, whiskers (1.5 times the interquartile range) and the outliers of the samples, and the points in the plot area represent different replicates of the corresponding treatment. Boxplots sharing one or more identical letters are not significantly different based on Tukey’s *post hoc* test. Statistics are shown in **Table 1**.

Plant biomass – Like plant height, total plant biomass was significantly enhanced by additional water (+38.0%), and fertilizer (9.0%) supply, and their joint effects were strongly synergistic (+234%, **Figure 2D**, **Table 1**, W × F interaction, p < 0.001). The same was observed for individual leaf, stem and root biomass (**Table 1**, **Figures 2A–C**). Effects of AMF depended on water conditions. Overall, inoculation with AMF reduced leaf, stem and total biomass, but this effect was only observed in plants that did not receive additional water supply (**Table 1**, M × W, p < 0.05, **Figures 2A, B, D**). Notably, when mycorrhizal effects were tested separately under each of the four environmental conditions, biomass reductions were only observed for plant stems under low fertilizer and soil phosphorus conditions (paired t-test, t=5.32, p < 0.05). Contrary to expectation, the benefits of AMF inoculation were thus not more pronounced at lower soil water and nutrient conditions. Root biomass was not influenced by inoculation with AMF (**Table 1**, **Figure 2C**).

### Plant functional traits

**Figure 3:**
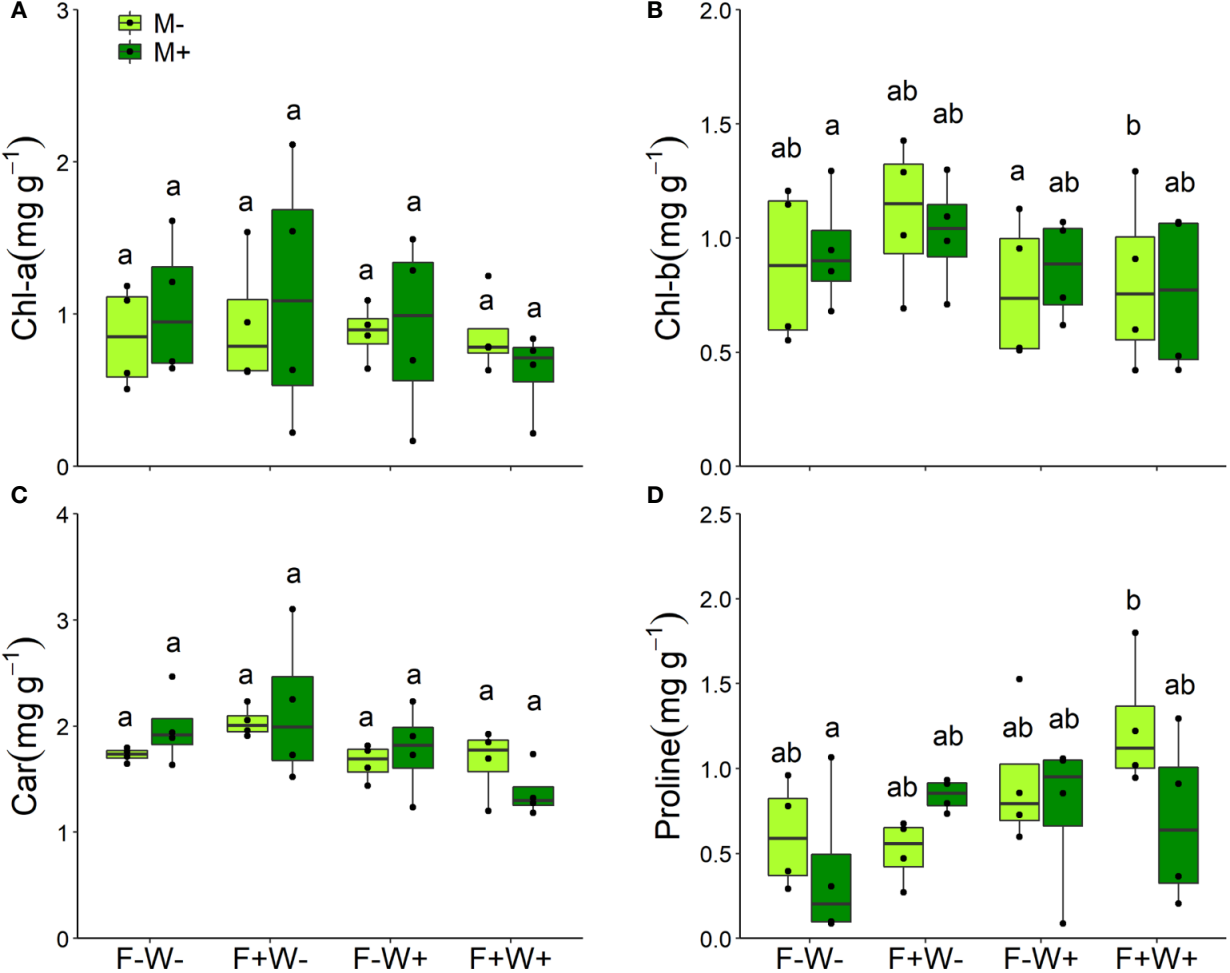
Contents of chlorophyll a (Chl-a, **A**), chlorophyll b (Chl-b, **B**), carotenoids **(C)** and proline **(D)** in the leaves of *Artemisia ordosica* plants that were inoculated with the AM fungal species *Funneliformis mosseae* (M+) or not (M-) and grown under four different combinations of fertilization (F-: no fertilization; F+: fertilization) and water addition (W-: low soil water; W+: high soil water) treatments. Light symbols: plants received no AMF; dark symbols: plants inoculated with AMF. The boxplot shows the 25% and 75% quartiles, the median, whiskers (1.5 times the interquartile range) and the outliers of the samples, and the points in the plot area represent different replicates of the corresponding treatment. Boxplots sharing one or more identical letters are not significantly different based on Tukey’s *post hoc* test. Statistics are shown in **Table 1**.

Photosynthetic and physiological traits – Leaf chlorophyll b, carotenoid, and proline contents were predominantly affected by water treatment. On average, proline content was higher but chlorophyll b and carotenoid contents were lower in leaves of plants that received additional water, whereas leaf chlorophyll a content was not affected by water treatment (**Table 1**, all p<0.01, **Figures 3A–D**). The reduction in leaf chlorophyll b content by additional water occurred only in plants that also received fertilization (**Table 1**, F × W, p<0.05, **Figure 3B**). When additional fertilizer was supplied, leaf proline content was enhanced by AMF under low water conditions, but reduced by AMF under high water conditions (AMF × F × W interaction, P < 0.05, **Table 1**, **Figure 3D**).

**Figure 4:**
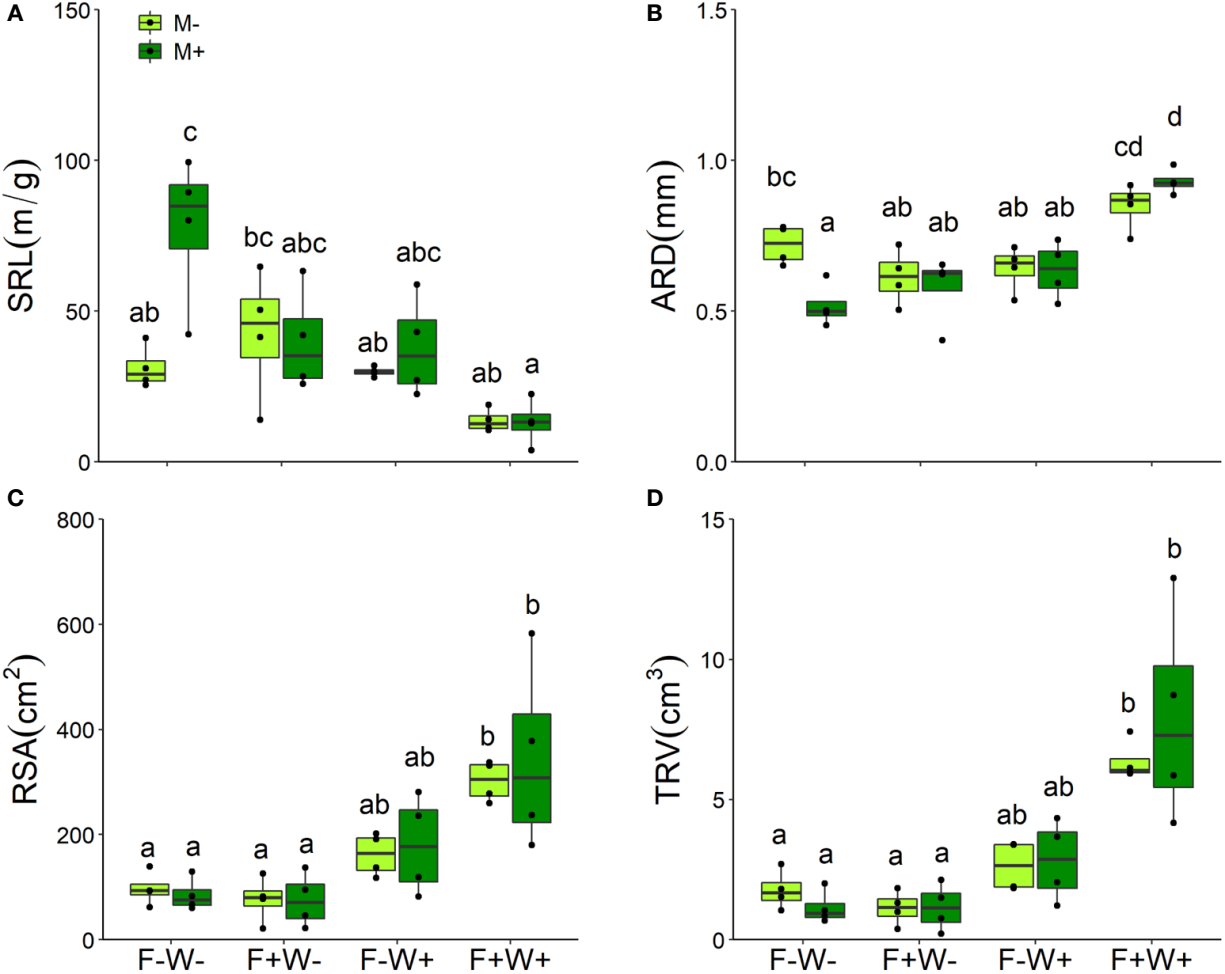
Specific root length (SRL, **A**), averaged root diameter (ARD, **B**), root surface area (RSA, **C**), and total root volume (TRV, **D**) of *Artemisia ordosica* plants that were inoculated with the AM fungal species *Funneliformis mosseae* (M+) or not (M-) and grown under four different combinations of fertilization (F-: no fertilization; F+: fertilization) and water addition (W-: low soil water; W+: high soil water) treatments. Light symbols: plants received no AMF; dark symbols: plants inoculated with AMF. The boxplot shows the 25% and 75% quartiles, the median, whiskers (1.5 times the interquartile range) and the outliers of the samples, and the points in the plot area represent different replicates of the corresponding treatment. Boxplots sharing one or more identical letters are not significantly different based on Tukey’s *post hoc* test. Statistics are shown in **Table 2**.

Root traits – Among the measured plant root traits, SRL was decreased but ARD was increased by fertilization (**Table 1**, **Figures 4A, B**). All measured root traits were significantly affected by water addition. But whereas water addition reduced SRL, it enhanced ARD, RSA and TRV. Effects of water addition were stronger in plants that were not fertilized than in fertilized plants (W × F interactions, all p < 0.01, **Table 1**, **Figures 4A–D**). SRL was enhanced by AMF, but this only occurred when plants did not receive fertilizer supply (**Table 1**, **Figure 4A**). The other root traits, including ARD, RSA and TRV were not affected by inoculation with AMF (**Table 1**, **Figures 4A–D**) except for ARD that was reduced by mycorrhizal inoculation under low fertilization and water conditions but enhanced by inoculation under high fertilization and water conditions (**Table 1**, **Figure 4B**).

**Figure 5:**
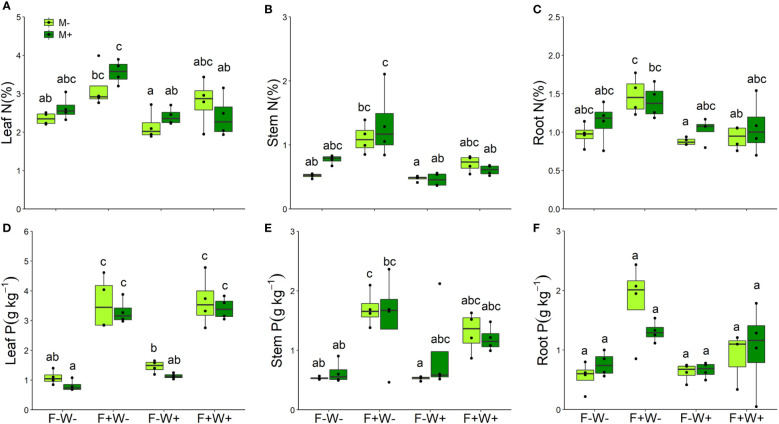
Nitrogen **(A–C)** and phosphorus **(D–F)** concentrations of different plant tissues of *Artemisia ordosica* that were inoculated with the AM fungal species *Funneliformis mosseae* (M+) or not (M-) and grown under four different combination of fertilization (F-: no fertilization; F+: fertilization) and water addition (W-: low soil water; W+: high soil water) treatments. Light symbols: plants received no AMF; dark symbols: plants inoculated with AMF *Funneliformis mosseae*. The boxplot shows the 25% and 75% quartiles, the median, whiskers (1.5 times the interquartile range) and the outliers of the samples, and the points in the plot area represent different replicates of the corresponding treatment. Boxplots sharing one or more identical letters are not significantly different based on Tukey’s *post hoc* test. Statistics are shown in **Table 2**.

Leaf nutritional traits – Concentrations of nitrogen in leaves, stems and roots were strongly enhanced by soil fertilization but reduced by water addition (**Table 2**, **Figures 5A–C**). Especially in roots, effects of fertilization on tissue N concentrations were stronger under low water conditions than under additional water supply (W × F interaction, p < 0.05, **Table 2**, **Figures 5A–C**). For the concentrations of phosphorus in leaves, stems and roots a similar pattern was observed. These were significantly enhanced by fertilization (**Table 2**, **Figures 5D–F**), but in leaves and roots this effect was more strongly observed if plants did not receive additional water (W × F interactions, p < 0.05, **Table 2**, **Figures 5D, F**). Surprisingly, leaf P concentration was overall significantly reduced by AMF (p < 0.01, **Table 2**, **Figure 5D**). Although the interaction between AMF and fertilizer treatment was not significant, the suppressive effect of AMF on leaf P tended to be stronger under low fertilization conditions (F-W-: t-test, t _[1, 3]_ = 4.89, p < 0.05; F-W+: t-test, t _[1, 3]_ = 2.42, p < 0.10) than under high fertilizer conditions (F+W- and F+W+: both p > 0.6).

**Table 2:** General Linear Mixed Models (GLMM) of the effects of inoculation with arbuscular mycorrhizal fungi (M), fertilization (F) and water addition (W) on plant nitrogen (N) and phosphorus (P) concentrations in leaves, stems and roots of *Artemisia ordosica* plants grown in the greenhouse, as well as generalized linear mixed model (“poisson” distribution) of the effects of M, F, and W treatments on the abundance of insect herbivore *Chrysolina aeruginosa* colonizing each plant in the field.

Source	df	N content			P content			No. *C. aeruginosa*
		Leaf	Stem	Root	Leaf	Stem	Root	
**M**	1	1.01	1.43	1.64	8.02^**^	0.15	0.02	11.0^***^
**F**	1	15.4^***^	44.4^***^	6.88^*^	389.4^***^	34.8^***^	12.5^**^	9.37^**^
**W**	1	11.9^**^	39.4^***^	12.6^**^	9.35^**^	0.08	5.10^*^	1.29
**M×F**	1	1.26	1.51	0.83	3.25	2.35	0.87	4.00^*^
**M×W**	1	1.58	6.60^*^	0.38	0.08	0.61	0.22	0.61
**F×W**	1	3.14	3.63	5.33^*^	6.60^*^	1.36	5.24^*^	9.44^**^
**M×P×W**	1	1.92	0.0.33	0.30	0.03	0.03	1.51	0.26

F values for N and P content, and χ^2^ values for herbivore abundance are shown in the table and those in bold indicate a significant treatment effect (P < 0.05). ^*^P < 0.05, ^**^P < 0.01, ^***^P < 0.001; df, numerator degrees of freedom. There were 4 replicates for data on plant N and P contents, and 8 replicates for data on number of *C. aeruginosa* in each of AMF, F, and W treatments.

### Herbivore abundance on plants transferred to the field

**Figure 6:**
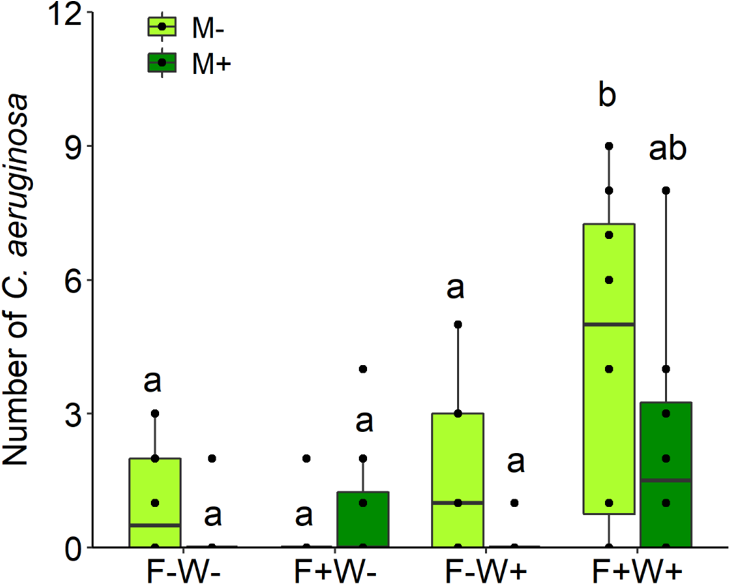
Abundance of the insect species *Chrysolina aeruginosa* on the aerial tissues of *Artemisia ordosica* that were inoculated with the AM fungal species *Funneliformis mosseae* (AMF+) or not (AMF-) and grown under four different combination of fertilization (F-: no fertilization; F+: fertilization) and water addition (W-: low soil water; W+: high soil water) treatments. Light symbols: plants received no AMF; dark symbols: plants inoculated with AMF *Funneliformis mosseae*. The boxplot shows the 25% and 75% quartiles, the median, whiskers (1.5 times the interquartile range) and the outliers of the samples, and the points in the plot area represent different replicates of the corresponding treatment. Boxplots sharing one or more identical letters are not significantly different based on Tukey’s *post hoc* test. Statistics are shown in **Table 2**.

Abundance of the herbivore *C. aeruginosa* was significantly lower on plants inoculated by AMF than on non-mycorrhizal plants, but this effect was only significant and stronger on non-fertilized plants (mean ± s.e. in insect abundance; AMF: 0.19 ± 0.14; non-AMF: 1.31 ± 0.38; t _[1, 15]_ = 2.70, p < 0.05) than on fertilized plants (AMF: 1.56 ± 0.57; non-AMF: 2.47 ± 0.87; t _[1, 15]_ = 1.10, p = 0.29), resulting in a significant two-way interactions between AMF and fertilization treatment (**Table 2**, M × F: χ^2^ = 4.00, p = 0.045, **Figure 6**). In contrast, herbivore abundance was overall higher on plants that had been fertilized, but only when these plants had also received additional watering (**Table 2**, F × W: χ^2^ = 9.44, p = 0.002, **Figure 6**). These treatment effects were not mediated by plant height (a proxy of plant size) at the time of measurement since inclusion of plant height as a covariate in the analyses (χ^2^ = 0.72, p = 0.397) did not alter the significance of the effects of AMF, fertilizer and watering treatments.

## Discussion

Our study examined the role of arbuscular mycorrhizal fungi in modifying plant traits related to growth, physiology and defense, in particular to what extent the role of AMF depends on soil water and phosphorus supply. We found that AMF inoculation repressed plant growth especially under low soil water and nutrient conditions. On the other hand, AMF tended to reduce the incidence of an important herbivorous insect under low soil fertilization conditions and thus alleviate potential damage under adverse environmental conditions, which could have been mediated by the lower leaf P content of AMF-inoculated plants.

### Effects of AMF, soil water and soil P on plant growth

Recent years witness an increasing number of studies recognizing that the outcome of plant-AMF interactions often show a continuum ranging from mutualism to parasitism, depending on the context in which the interactions occur (Hoeksema et al., 2010; Johnson et al., 2015; Jin et al., 2017; Qu et al., 2021). However, these results were mostly obtained from studies using short-lived herbaceous plant species and commercial AMF strains as a model system, and it is yet unclear whether these results apply to plants of different life forms. In the current study, a shrub species, *A. ordosica* was used to measure its responses to AMF under contrasting soil water and soil fertilization conditions. Our results show that inoculation of *A. ordosica* with a strain of the AM fungus *F. mosseae* that was isolated from a comparable sandy, low precipitation habitat as the collection site of the host plant seeds, overall reduced plant height, and the reduction consistently increased over time as plants grew. This result may relate to the overall low root colonization (5% on average) by the mycorrhizal strain, indicating a potentially limited opportunity for beneficial C-P trade in this symbiotic combination (Graham and Abbott, 2000). Such low colonization rate may result in low transfer of water and nutrients to the plant. If, even at low levels of colonization, the AMF would still suppress the plant’s direct pathway of P uptake through its own root system, this cost could outweigh the benefits of the indirect pathway provided by mycorrhizal extra-radical mycelia (Smith et al., 2011) and jeopardize the beneficial C-P trade traditionally hypothesized for mycorrhizal symbiosis (Grace et al., 2009; Johnson, 2010). The reason for the overall low root colonization of *A. ordosica* in our experiment is unknown, but low colonization rates of *F. mosseae* have been observed in other plant species as well. For instance, average colonization percentages of a single *F. mosseae* strain varied between 2.6 and 27.0% across a range of tomato cultivars (Steinkellner et al., 2012). Interestingly, the cultivar with the lowest colonization percentage nevertheless showed a significant 30% increase in root dry weight in response to inoculation, whereas the root weight of the cultivar with the highest colonization percentage was not affected by *F. mosseae*. This indicates that, whatever the reason for low colonization is, even low percentages of colonization can significantly affect plant performance, and that the magnitude of AMF effects is not necessarily related to percentage of colonization. It should be noted that despite the low colonization in M+ plants and the unexpected presence of colonization in M- plants, possibly due to cross-contamination, AMF colonization percentages were still significantly higher in M+ plants than in M- plants, indicating that the validity of AMF treatments in our study was not compromised.

Given that *F. mosseae* was one of the dominant AMF species in the study area and that the *F. mosseae* isolate used in the current study originated from a region similar to the region where the plant species *A. ordosica* is commonly found, potential incompatibility between the two partners due to an ecological mismatch should not be a reason for the observed mycorrhizal growth depression (Jin et al., 2017; Řezáčová et al., 2017). Surprisingly, the mycorrhizal plant growth reduction was most strongly observed under low soil water and phosphorus conditions. This is in contrast with other studies showing that AMF inoculation usually alleviates adverse impacts of drought stress (Duc et al., 2018; Liu et al., 2021; Jongen et al., 2022). The mechanism underlying this drought-induced mycorrhizal plant growth reduction is unknown, but it may relate to the observed interactive negative effects of mycorrhizae and drought on root traits, such as specific root length (SRL) or average root diameter (ARD) (Chen et al., 2016). Plants with higher SRL or lower ARD as observed in the current study tend to have greater plasticity in water and nutrient uptake, but they often show less mycorrhizal dependency, and this may be the reason for the mycorrhizal plant growth depression (Eissenstat, 1992). Even more striking was the observation that the mycorrhizal growth reduction tended to be stronger under non-fertilized than under fertilized conditions. Numerous studies have shown that the mycorrhizal growth response becomes more beneficial for plants at lower nutrient levels (e.g. Vogelsang et al., 2006; Klironomos et al., 2011; Johnson et al., 2015), although exceptions have been reported (Püschel et al., 2016; Raya-Hernandez et al., 2020). Perhaps competition for nutrients between AMF and plants occurred under these experimental conditions, which may have aggravated the AMF-induced plant growth reduction (Li et al., 2008).

The mycorrhizal growth reduction was not only observed for plant height but also for plant biomass. Our results showed that inoculation of AMF significantly reduced plant biomass, and, as observed for plant height, the strength of the AMF-induced reduction in plant biomass production depended on soil water and nutrient conditions. Leaf and stem biomass was more strongly suppressed by AMF inoculation under low water or nutrient supply than under more favorable conditions. In addition to the potential competition between AMF and host plants as previously suggested, an alternative explanation for this observation may be that the AMF strain was not adapted to perform optimally with the host under the low water and nutrient conditions since the site of origin of AMF strain used in this study has a higher mean annual precipitation than the experimental region from which the host plants were collected, mimicked by the low water and nutrient conditions. On the other hand, it is interesting to notice that root biomass was not influenced by AMF inoculation. This result indicates that the plant growth response to AMF inoculation was more sensitive to soil heterogeneity in the shoots than in the roots, suggesting the dependence of AMF-host interactions on environmental conditions may differ among plant functional organs (Roesti et al., 2006; Yang et al., 2016).

Many studies have shown that the photosynthetic capacity of mycorrhizal plants is often higher than that of non-mycorrhizal plants (Liu et al., 2015; Zhang et al., 2018; Balestrini et al., 2020). Such improvements can be incurred by enhanced chlorophyll contents, photosynthetic rate and transpiration rate in plants following AMF colonization (Chandrasekaran et al., 2019). However, in our study we did not observe significant effects of AMF inoculation on the concentration of chlorophyll a, chlorophyll b or carotenoids in *A. ordosica*. This result is in contrast to the results of Zhu et al. (2011) who showed that all these photosynthetic traits were higher in maize colonized by the AMF species *Glomus etunicatum* than in non-mycorrhizal plants. The lack of AMF effects on photosynthesis-related traits in our experiment may be explained by the extremely low AMF colonization rate of *A. ordosica* that might be insufficient to systemically induce changes in the plant’s light harvesting capacity (Evelin et al., 2009). AMF inoculation also did not significantly enhance leaf levels of the osmolyte proline. Osmolytes are often synthesized under water stress in order to maintain osmotic balance (Furlan et al., 2020), but this was not observed in our experiment. The reasons for this unexpected result are unknown, but it may be related to the unfavorable growing environment in the greenhouse, e.g. low intensity of light, that can prevent stress-induced plant responses (Lin et al., 2019).

### Effects of AMF, soil water and soil P on abundance of colonizing herbivores

In this study, we exposed AMF inoculated and control plants grown under different soil water and nutrient conditions to natural herbivores. The relatively lower abundance of the herbivore species *C. aeruginosa* on mycorrhizal than on non-mycorrhizal plants when grown under low fertilizer conditions suggests a reduced attractiveness of AMF-inoculated plants for this insect species. This result complies with the finding that AMF generally induce resistance to leaf chewing herbivores (Pozo and Azcon-Aguilar, 2007; Koricheva et al., 2009; Jung et al., 2012; Meier and Hunter, 2018; Jiang et al., 2021). However, this effect was only observed in plants grown under low soil P, illustrating the context-dependency of the effects of AMF on plant-herbivore interactions. This observation is in line with other studies. For instance, Wang et al. (2020) showed that only in low-nutrient soils AMF colonization was high but aphid infestation was low. Not only soil P content, but also other environmental factors such as light were reported to influence the effects of mycorrhizal inoculation on plant-insect interactions (Qu et al., 2021). Several mechanisms have been proposed to underlie plant defense responses to AMF inoculation, including priming effects or enhanced production of defensive metabolites (Koricheva et al., 2009). In our study, the observed lower incidence of *C. aeruginosa* on AMF-inoculated plants under low fertilization conditions could have been related to the observed lower P concentrations in mycorrhizal plant leaves under these conditions. Even though AMF incurred only a modest reduction in leaf P under these conditions, overall leaf P levels under these conditions were very low, so that further reductions may have had a significant impact on herbivore preference; lower leaf P concentrations generally represent a lower diet quality for herbivores (Real-Santillan et al., 2019). Interestingly, under higher fertilization levels, the preference of the herbivore for non-mycorrhizal over mycorrhizal plants disappeared. Since the overall levels of leaf phosphorus were much higher under these conditions, this could indicate that once a threshold P concentration of leaves has been reached, the preference of *C. aeruginosa* is no longer driven by P-demand, but by other stoichiometric resource requirements such as a higher N demand under conditions where P is no longer limiting, or by other factors not significantly affected by mycorrhizal inoculation. Leaf nitrogen is another important leaf trait that affects leaf nutritional quality for herbivores either through provisioning of primary metabolites or through N-based secondary metabolite production, but leaf N in our experiment was not significantly affected by mycorrhizal inoculation. An alternative explanation for the lower abundance of the chrysomelid beetle on AMF-inoculated plants grown under low soil fertilization could be the AMF-induced reduction in leaf and stem biomass under these conditions. However, inclusion of plant height as a covariate in the analysis did not account for any variation in herbivore abundance, so this idea is not supported by our data.

## Conclusion

In the current study we surprisingly found that effects of AMF on plant growth do not follow the hypothesized pattern that low availability of soil water and nutrient favor the functionality of host-AMF interactions. Mycorrhizal inoculation caused a growth depression in plant height and biomass especially under drought and low-nutrient conditions. Furthermore, we observed that AMF reduced the abundance of a specialist herbivore on plants grown under low soil fertilizer levels, which might be associated with the lower leaf P concentrations in these plants. We thus conclude that plant responses to AMF inoculation may differ in terms of the traits measured and the types of environmental factors the plants experience.

## Data availability statement

The raw data supporting the conclusions of this article will be made available by the authors, without undue reservation.

## Author contributions

LQ and AB conceived the idea of this study. LQ, ZW and MG performed the experiments, and MW, LQ and AB analyzed the data and wrote the manuscript. All authors contributed to the article and approved the submitted version.

## References

[B1] Adeyemi N. O. Atayese M. O. Sakariyawo O. S. Azeez J. O. Ridwan M. (2021). Arbuscular mycorrhizal fungi species differentially regulate plant growth, phosphorus uptake and stress tolerance of soybean in lead contaminated soil. J. Plant Nutr. 44, 1633–1648. doi: 10.1080/01904167.2021.1871748

[B2] Balestrini R. Brunetti C. Chitarra W. Nerva L. (2020). Photosynthetic traits and nitrogen uptake in crops: Which is the role of arbuscular mycorrhizal fungi? Plants-Basel 9, 1105. doi: 10.3390/plants9091105 32867243 PMC7570035

[B3] Bates L. S. (1973). Rapid determination of free proline for water stress studies. Plant Soil 39, 205–207. doi: 10.1007/BF00018060

[B4] Bates D. Machler M. Bolker B. M. Walker S. C. (2015). Fitting linear mixed-effects models using lme4. J. Stat. Software 67, 1–48. doi: 10.18637/jss.v067.i01

[B5] Begum N. Akhtar K. Ahanger M. A. Iqbal M. Wang P. P. Mustafa N. S.. (2021). Arbuscular mycorrhizal fungi improve growth, essential oil, secondary metabolism, and yield of tobacco (*Nicotiana tabacum* l.) under drought stress conditions. Environ. Sci. pollut. Res. 28, 45276–45295. doi: 10.1007/s11356-021-13755-3 33860891

[B6] Begum N. Qin C. Ahanger M. A. Raza S. Khan M. I. Ashraf M.. (2019). Role of arbuscular mycorrhizal fungi in plant growth regulation: Implications in abiotic stress tolerance. Front. Plant Sci. 10. doi: 10.3389/fpls.2019.01068 PMC6761482 31608075

[B7] Bernaola L. Stout M. J. (2019). Effects of arbuscular mycorrhizal fungi on rice-herbivore interactions are soil-dependent. Sci. Rep. 9, 14037. doi: 10.1038/s41598-019-50354-2 31575889 PMC6773947

[B8] Bierman B. Linderman R. G. (1981). Quantifying vesicular-arbuscular mycorrhizae: A proposed method towards standardization. New Phytol. 87, 63–67. doi: 10.1111/j.1469-8137.1981.tb01690.x

[B9] Bonfante P. Genre A. (2010). Mechanisms underlying beneficial plant-fungus interactions in mycorrhizal symbiosis. Nat. Commun. 1, 48. doi: 10.1038/ncomms1046 20975705

[B10] Boyer L. R. Brain P. Xu X. M. Jeffries P. (2015). Inoculation of drought-stressed strawberry with a mixed inoculum of two arbuscular mycorrhizal fungi: effects on population dynamics of fungal species in roots and consequential plant tolerance to water deficiency. Mycorrhiza 25, 215–227. doi: 10.1007/s00572-014-0603-6 25186649

[B11] Bucking H. Kafle A. (2015). Role of arbuscular mycorrhizal fungi in the nitrogen uptake of plants: current knowledge and research gaps. Agronomy-Basel 5, 587–612. doi: 10.3390/agronomy5040587

[B12] Chandrasekaran M. Chanratana M. Kim K. Seshadri S. Sa T. (2019). Impact of arbuscular mycorrhizal fungi on photosynthesis, water status, and gas exchange of plants under salt stress - a meta-analysis. Front. Plant Sci. 10. doi: 10.3389/fpls.2019.00457 PMC6476944 31040857

[B13] Chen W. L. Koide R. T. Adams T. S. DeForest J. L. Cheng L. Eissenstat D. M. (2016). Root morphology and mycorrhizal symbioses together shape nutrient foraging strategies of temperate trees. Proc. Natl. Acad. Sci. U.S.A. 113, 8741–8746. doi: 10.1073/pnas.1601006113 27432986 PMC4978252

[B14] Chitarra W. Maserti B. Gambino G. Guerrieri E. Balestrini R. (2016). Arbuscular mycorrhizal symbiosis-mediated tomato tolerance to drought. Plant Signal. Behav. 11, e1197468. doi: 10.1080/15592324.2016.1197468 27359066 PMC4991350

[B15] Cozzolino V. De Martino A. Nebbioso A. Di Meo V. Salluzzo A. Piccolo A. (2016). Plant tolerance to mercury in a contaminated soil is enhanced by the combined effects of humic matter addition and inoculation with arbuscular mycorrhizal fungi. Environ. Sci. pollut. Res. 23, 11312–11322. doi: 10.1007/s11356-016-6337-6 26931658

[B16] Delavaux C. S. Smith-Ramesh L. M. Kuebbing S. E. (2017). Beyond nutrients: A meta-analysis of the diverse effects of arbuscular mycorrhizal fungi on plants and soils. Ecology 98, 2111–2119. 28500779 10.1002/ecy.1892

[B17] Díaz A. S. L. Macheda D. Saha H. Ploll U. Orine D. Biere A. (2021). Tackling the context-dependency of microbial-induced resistance. Agronomy 11, 1293. doi: 10.3390/agronomy11071293

[B18] Dowarah B. Gill S. S. Agarwala N. (2022). Arbuscular mycorrhizal fungi in conferring tolerance to biotic stresses in plants. J. Plant Growth Regul. 41, 1429–1444.

[B19] Duc N. H. Csintalan Z. Posta K. (2018). Arbuscular mycorrhizal fungi mitigate negative effects of combined drought and heat stress on tomato plants. Plant Physiol. Biochem. 132, 297–307. doi: 10.1016/j.plaphy.2018.09.011 30245343

[B20] Eissenstat D. M. (1992). Costs and benefits of constructing roots of small diameter. J. Plant Nutr. 15, 763–782.

[B21] Evelin H. Devi T. S. Gupta S. Kapoor R. (2019). Mitigation of salinity stress in plants by arbuscular mycorrhizal symbiosis: current understanding and new challenges. Front. Plant Sci. 10. doi: 10.3389/fpls.2019.00470 PMC6473083 31031793

[B22] Evelin H. Kapoor R. Giri B. (2009). Arbuscular mycorrhizal fungi in alleviation of salt stress: A review. Ann. Bot. 104, 1263–1280. doi: 10.1093/aob/mcp251 19815570 PMC2778396

[B23] Ferrol N. Azcon-Aguilar C. Perez-Tienda J. (2019). Review: Arbuscular mycorrhizas as key players in sustainable plant phosphorus acquisition: An overview on the mechanisms involved. Plant Sci. 280, 441–447. doi: 10.1016/j.plantsci.2018.11.011 30824024

[B24] Fox J. Weisberg S. (2019). An R companion to applied regression, third edition (Thousand Oaks, California: SAGE Publications, Inc.).

[B25] Furlan A. L. Bianucci E. Giordano W. Castro S. Becker D. F. (2020). Proline metabolic dynamics and implications in drought tolerance of peanut plants. Plant Physiol. Biochem. 151, 566–578. doi: 10.1016/j.plaphy.2020.04.010 32320942

[B26] Grace E. J. Cotsaftis O. Tester M. Smith F. A. Smith S. E. (2009). Arbuscular mycorrhizal inhibition of growth in barley cannot be attributed to extent of colonization, fungal phosphorus uptake or effects on expression of plant phosphate transporter genes. New Phytol. 181, 938–949. doi: 10.1111/j.1469-8137.2008.02720.x 19140934

[B27] Graham J. H. Abbott L. K. (2000). Wheat responses to aggressive and non-aggressive arbuscular mycorrhizal fungi. Plant Soil 220, 207–218. doi: 10.1023/A:1004709209009

[B28] Hoeksema J. D. Chaudhary V. B. Gehring C. A. Johnson N. C. Karst J. Koide R. T.. (2010). A meta-analysis of context-dependency in plant response to inoculation with mycorrhizal fungi. Ecol. Lett. 13, 394–407. doi: 10.1111/j.1461-0248.2009.01430.x 20100237

[B29] Intergovernmental Panel on Climate Change (2018). IPCC special report on the impacts of global warming of 1.5 ℃: Summary for policy makers (Incheon, South Korea).

[B30] Irankhah S. Chitarra W. Nerva L. Antoniou C. Lumini E. Volpe V.. (2020). Impact of an arbuscular mycorrhizal fungal inoculum and exogenous MeJA on fenugreek secondary metabolite production under water deficit. Environ. Exp. Bot. 176. doi: 10.1016/j.envexpbot.2020.104096

[B31] Jiang D. Tan M. T. Wu S. Zheng L. Wang Q. Wang G. R.. (2021). Defense responses of arbuscular mycorrhizal fungus-colonized poplar seedlings against gypsy moth larvae: A multiomics study. Hortic. Res. 8, 245. doi: 10.1038/s41438-021-00671-3 34848684 PMC8632881

[B32] Jin L. Wang Q. Wang Q. Wang X. J. Gange A. C. (2017). Mycorrhizal-induced growth depression in plants. Symbiosis 72, 81–88. doi: 10.1007/s13199-016-0444-5

[B33] Johnson N. C. (2010). Resource stoichiometry elucidates the structure and function of arbuscular mycorrhizas across scales. New Phytol. 185, 631–647. doi: 10.1111/j.1469-8137.2009.03110.x 19968797

[B34] Johnson N. C. Wilson G. W. T. Wilson J. A. Miller R. M. Bowker M. A. (2015). Mycorrhizal phenotypes and the law of the minimum. New Phytol. 205, 1473–1484. doi: 10.1111/nph.13172 25417818

[B35] Jongen M. Albadran B. Beyschlag W. Unger S. (2022). Can arbuscular mycorrhizal fungi mitigate drought stress in annual pasture legumes? Plant Soil 472, 295–310. doi: 10.1007/s11104-021-05233-z

[B36] Jung S. C. Martinez-Medina A. Lopez-Raez J. A. Pozo M. J. (2012). Mycorrhiza-induced resistance and priming of plant defenses. J. Chem. Ecol. 38, 651–664. doi: 10.1007/s10886-012-0134-6 22623151

[B37] Kaur S. Campbell B. J. Suseela V. (2022). Root metabolome of plant–arbuscular mycorrhizal symbiosis mirrors the mutualistic or parasitic mycorrhizal phenotype. New Phytol. 234, 672–687. doi: 10.1111/nph.17994 35088406

[B38] Kaur S. Suseela V. (2020). Unraveling arbuscular mycorrhiza-induced changes in plant primary and secondary metabolome. Metabolites 10, 335. doi: 10.3390/metabo10080335 32824704 PMC7464697

[B39] Klironomos J. Zobel M. Tibbett M. Stock W. D. Rillig M. C. Parrent J. L.. (2011). Forces that structure plant communities: Quantifying the importance of the mycorrhizal symbiosis. New Phytol. 189, 366–370. doi: 10.1111/j.1469-8137.2010.03550.x 21058952

[B40] Koricheva J. Gange A. C. Jones T. (2009). Effects of mycorrhizal fungi on insect herbivores: A meta-analysis. Ecology 90, 2088–2097. doi: 10.1890/08-1555.1 19739371

[B41] Konvalinková T. Jansa J. (2016). Lights off for arbuscular mycorrhiza: On its symbiotic functioning under light deprivation. Front. Plant Sci. 7. doi: 10.3389/fpls.2016.00782 PMC4893486 27375642

[B42] Kuznetsova A. Brockhoff P. B. Christensen R. H. B. (2017). lmerTest package: Tests in linear mixed effects models. J. Stat. Software 82, 1–26. doi: 10.18637/jss.v082.i13

[B43] Lichtenthaler K. Welburn A. R. (1983). Determination of total carotenoids and chlorophylls a and b of leaf extracts in different solvents. Biochem. Soc Trans. 11, 591–592. doi: 10.1042/bst0110591

[B44] Lin J. H. Zhang R. Hu Y. Y. Song Y. Hanninen H. Wu J. S. (2019). Interactive effects of drought and shading on torreya grandis seedlings: Physiological and growth responses. Trees-Struct. Funct. 33, 951–961. doi: 10.1007/s00468-019-01831-8

[B45] Li H. Y. Smith F. A. Dickson S. Holloway R. E. Smith S. E. (2008). Plant growth depressions in arbuscular mycorrhizal symbioses: Not just caused by carbon drain? New Phytol. 178, 852–862. doi: 10.1111/j.1469-8137.2008.02410.x 18346106

[B46] Liu L. Li D. Ma Y. L. Shen H. T. Zhao S. M. Wang Y. F. (2021). Combined application of arbuscular mycorrhizal fungi and exogenous melatonin alleviates drought stress and improves plant growth in tobacco seedlings. J. Plant Growth Regul. 40, 1074–1087. doi: 10.1007/s00344-020-10165-6

[B47] Liu C. Y. Zhang F. Zhang D. J. Srivastava A. K. Wu Q. S. Zou Y. N. (2018). Mycorrhiza stimulates root-hair growth and IAA synthesis and transport in trifoliate orange under drought stress. Sci. Rep. 8, 1978. doi: 10.1038/s41598-018-20456-4 29386587 PMC5792640

[B48] Liu T. Sheng M. Wang C. Y. Chen H. Li Z. Tang M. (2015). Impact of arbuscular mycorrhizal fungi on the growth, water status, and photosynthesis of hybrid poplar under drought stress and recovery. Photosynthetica 53, 250–258.

[B49] Li Z. Wu N. Liu T. Chen H. Tang M. (2015). Effect of arbuscular mycorrhizal inoculation on water status and photosynthesis of *Populus cathayana* males and females under water stress. Physiol. Plantarum. 155, 192–204. doi: 10.1111/ppl.12336 25720810

[B50] Li S. L. Yu F. H. Werger M. J. A. Dong M. Zuidema P. A. (2011). Habitat-specific demography across dune fixation stages in a semi-arid sandland: Understanding the expansion, stabilization and decline of a dominant shrub. J. Ecol. 99, 610–620. doi: 10.1111/j.1365-2745.2010.01777.x

[B51] Mathur S. Sharma M. P. Jajoo A. (2018). Improved photosynthetic efficacy of maize (*Zea mays*) plants with arbuscular mycorrhizal fungi (AMF) under high temperature stress. J. Photochem. Photobiol. B. Biol. 180, 149–154. doi: 10.1016/j.jphotobiol.2018.02.002 29425887

[B52] Meier A. R. Hunter M. D. (2018). Arbuscular mycorrhizal fungi mediate herbivore-induction of plant defenses differently above and belowground. Oikos 127, 1759–1775. doi: 10.1111/oik.05402

[B53] Mickan B. S. Hart M. Solaiman Z. M. Renton M. Siddique K. H. M. Jenkins S. N.. (2021). Arbuscular mycorrhizal fungus-mediated interspecific nutritional competition of a pasture legume and grass under drought-stress. Rhizosphere 18, 100349. doi: 10.1016/j.rhisph.2021.100349

[B54] Nath M. Bhatt D. Prasad R. Gill S. S. Anjum N. A. Tuteja N. (2016). Reactive oxygen species generation-scavenging and signaling during plant - arbuscular mycorrhizal and piriformospora indica interaction under stress condition. Front. Plant Sci. 7. doi: 10.3389/fpls.2016.01574 PMC5073151 27818671

[B55] Nobre C. P. Huertas O. C. T. H. Tardin J. R. F. Saggin Junior O. J. Fonseca H. M. A. C. Berbara R. L. L. (2013). Biostimulation of inoculation with *Glomus proliferum* and application of humic acid in the *in vitro* growth of *Lunularia cruciate.* acta bot. Brasilica 27, 773–778. doi: 10.1590/S0102-33062013000400017

[B56] Osakabe Y. Osakabe K. Shinozaki K. Tran L. S. P. (2014). Response of plants to water stress. Front. Plant Sci. 5. doi: 10.3389/fpls.2014.00086 PMC3952189 24659993

[B57] Pasbani B. Salimi A. Aliasgharzad N. Hajiboland R. (2020). Colonization with arbuscular mycorrhizal fungi mitigates cold stress through improvement of antioxidant defense and accumulation of protecting molecules in eggplants. Sci. Hortic. 272, 109575. doi: 10.1016/j.scienta.2020.109575

[B58] Pozo M. J. Azcon-Aguilar C. (2007). Unraveling mycorrhiza-induced resistance. Curr. Opin. Plant Biol. 10, 393–398. doi: 10.1016/j.pbi.2007.05.004 17658291

[B59] Puschel D. Janouskova M. Hujslova M. Slavikova R. Gryndlerova H. Jansa J. (2016). Plant-fungus competition for nitrogen erases mycorrhizal growth benefits of *Andropogon gerardii* under limited nitrogen supply. Ecol. Evol. 6, 4332–4346. doi: 10.1002/ece3.2207 27386079 PMC4930984

[B60] Qian W. He X. (2009). Diversity of arbuscular mycorrhizal fungi associated with a desert plant artemisia ordosica. Biodivers. Sci. 17, 506–511. doi: 10.3724/SP.J.1003.2009.09020

[B61] Qu L. Y. Wang M. G. Biere A. (2021). Interactive effects of mycorrhizae, soil phosphorus, and light on growth and induction and priming of defense in *Plantago lanceolata* . Front. Plant Sci. 12. doi: 10.3389/fpls.2021.647372 PMC8021950 33833771

[B62] Raya-Hernandez A. I. Jaramillo-Lopez P. F. Lopez-Carmona D. A. Diaz T. Carrera-Valtierra J. A. Larsen J. (2020). Field evidence for maize-mycorrhiza interactions in agroecosystems with low and high p soils under mineral and organic fertilization. Appl. Soil Ecol. 149, 203511. doi: 10.1016/j.apsoil.2020.103511

[B63] R Core Development Team (2022). R: A language and environment for statistical computing. r found. stat. comput (Vienna, Austria). Available at: http://www.R-project.org/.

[B64] Real-Santillan R. O. del-Val E. Cruz-Ortega R. Contreras-Cornejo H. A. Gonzalez-Esquivel C. E. Larsen J. (2019). Increased maize growth and p uptake promoted by arbuscular mycorrhizal fungi coincide with higher foliar herbivory and larval biomass of the fall armyworm *Spodoptera frugiperda* . Mycorrhiza 29, 615–622. doi: 10.1007/s00572-019-00920-3 31724088

[B65] Řezáčová V. Slavíková R. Konvalinková T. Hujslová M. Gryndlerová H. Gryndler M.. (2017). Imbalanced carbon-for-phosphorus exchange between European arbuscular mycorrhizal fungi and non-native *Panicum* grasses - a case of dysfunctional symbiosis. Pedobiologia 62, 48–55. doi: 10.1016/j.pedobi.2017.05.004

[B66] Roesti D. Gaur R. Johri B. N. Imfeld G. Sharma S. Kawaljeet K.. (2006). Plant growth stage, fertiliser management and bio-inoculation of arbuscular mycorrhizal fungi and plant growth promoting rhizobacteria affect the rhizobacterial community structure in rain-fed wheat fields. soil biol. Biochem 38, 1111–1120. doi: 10.1016/j.soilbio.2005.09.010

[B67] Ruiz-Lozano J. M. Aroca R. Zamarreno A. M. Molina S. Andreo-Jimenez B. Porcel R.. (2016). Arbuscular mycorrhizal symbiosis induces strigolactone biosynthesis under drought and improves drought tolerance in lettuce and tomato. Plant Cell Environ. 39, 441–452. doi: 10.1111/pce.12631 26305264

[B68] Ruth B. Khalvati M. Schmidhalter U. (2011). Quantification of mycorrhizal water uptake *via* high-resolution on-line water content sensors. Plant Soil 342, 459–468. doi: 10.1007/s11104-010-0709-3

[B69] Saha H. Kaloterakis N. Harvey J. A. van der Putten W. H. Biere A. (2022). Effects of light quality on colonization of tomato roots by AMF and implications for growth and defense. Plants-Basel 11, 861. doi: 10.3390/plants11070861 35406841 PMC9002964

[B70] Schroeder M. S. Janos D. P. (2005). Plant growth, phosphorus nutrition, and root morphological responses to arbuscular mycorrhizas, phosphorus fertilization, and intraspecific density. Mycorrhiza 15, 203–216. doi: 10.1007/s00572-004-0324-3 15316886

[B71] Sharma E. Anand G. Kapoor R. (2017). Terpenoids in plant and arbuscular mycorrhiza-reinforced defence against herbivorous insects. Ann. Bot. 119, 791–801. doi: 10.1093/aob/mcw263 28087662 PMC5378189

[B72] She W. W. Bai Y. X. Zhang Y. Q. Qin S. G. Liu Z. Wu B. (2017). Plasticity in meristem allocation as an adaptive strategy of a desert shrub under contrasting environments. Front. Plant Sci. 8. doi: 10.3389/fpls.2017.01933 PMC5684672 29170676

[B73] Smith S. E. Facelli E. Pope S. Smith F. A. (2010). Plant performance in stressful environments: Interpreting new and established knowledge of the roles of arbuscular mycorrhizas. Plant Soil 326, 3–20. doi: 10.1007/s11104-009-9981-5

[B74] Smith F. A. Grace E. J. Smith S. E. (2009). More than a carbon economy: Nutrient trade and ecological sustainability in facultative arbuscular mycorrhizal symbioses. New Phytol. 182, 347–358. doi: 10.1111/j.1469-8137.2008.02753.x 19207688

[B75] Smith S. E. Jakobsen I. Gronlund M. Smith F. A. (2011). Roles of arbuscular mycorrhizas in plant phosphorus nutrition: Interactions between pathways of phosphorus uptake in arbuscular mycorrhizal roots have important implications for understanding and manipulating plant phosphorus acquisition. Plant Physiol. 156, 1050–1057. doi: 10.1104/pp.111.174581 21467213 PMC3135927

[B76] Smith S. E. Read D. J. (2008). Mycorrhizal symbiosis. 3rd Edition (London: Academic Press).

[B77] Song Y. Y. Ye M. Li C. Y. Wang R. L. Wei X. C. Luo S. M.. (2013). Priming of anti-herbivore defense in tomato by arbuscular mycorrhizal fungus and involvement of the jasmonate pathway. J. Chem. Ecol. 39, 1036–1044. doi: 10.1007/s10886-013-0312-1 23797931

[B78] Steinkellner S. Hage-Ahmed K. Garcia-Garrido J. M. Illana A. Ocampo J. A. Vierheilig H. (2012). A comparison of wild-type, old and modern tomato cultivars in the interaction with the arbuscular mycorrhizal fungus *Glomus mosseae* and the tomato pathogen *Fusarium oxysporum* f. sp lycopersici. Mycorrhiza 22 (3), 189–194. doi: 10.1007/s00572-011-0393-z 21674299

[B79] Ujvari G. Turrini A. Avio L. Agnolucci M. (2021). Possible role of arbuscular mycorrhizal fungi and associated bacteria in the recruitment of endophytic bacterial communities by plant roots. Mycorrhiza 31, 527–544. doi: 10.1007/s00572-021-01040-7 34286366 PMC8484141

[B80] Vannette R. L. Hunter M. D. Rasmann S. (2013). Arbuscular mycorrhizal fungi alter above- and below-ground chemical defense expression differentially among *Asclepias* species. Front. Plant Sci. 4. doi: 10.3389/fpls.2013.00361 PMC3776932 24065971

[B81] Vogelsang K. M. Reynolds H. L. Bever J. D. (2006). Mycorrhizal fungal identity and richness determine the diversity and productivity of a tallgrass prairie system. New Phytol. 172, 554–562. doi: 10.1111/j.1469-8137.2006.01854.x 17083685

[B82] Wang M. G. Bezemer T. M. van der Putten W. H. Biere A. (2015). Effects of the timing of herbivory on plant defense induction and insect performance in ribwort plantain (*Plantago lanceolata* l.) depend on plant mycorrhizal status. J. Chem. Ecol. 41, 1006–1017. doi: 10.1007/s10886-015-0644-0 26552915 PMC4670619

[B83] Wang C. Tian B. L. Yu Z. Z. Ding J. Q. (2020). Effect of different combinations of phosphorus and nitrogen fertilization on arbuscular mycorrhizal fungi and aphids in wheat. Insects 11, 365. doi: 10.3390/insects11060365 32545401 PMC7349843

[B84] Wu Q. S. He J. D. Srivastava A. K. Zou Y. N. Kuca K. (2019). Mycorrhizas enhance drought tolerance of citrus by altering root fatty acid compositions and their saturation levels. Tree Physiol. 39, 1149–1158. doi: 10.1093/treephys/tpz039 30957149

[B85] Xie K. Ren Y. H. Chen A. Q. Yang C. F. Zheng Q. S. Chen J.. (2022). Plant nitrogen nutrition: The roles of arbuscular mycorrhizal fungi. J. Plant Physiol. 269, 153591. doi: 10.1016/j.jplph.2021.153591 34936969

[B86] Yang H. S. Xu J. L. Guo Y. Koide R. T. Dai Y. J. Xu M. M.. (2016). Predicting plant response to arbuscular mycorrhizas: The role of host functional traits. Fungal Ecol. 20, 79–83. doi: 10.1016/j.funeco.2015.12.001

[B87] Zar J. H. (1999). Biostatistical analysis. 4th Edition (Upper Saddle River: Prentice Hall).

[B88] Zhang Z. S. Li X. R. Wang T. Wang X. P. Xue Q. W. Liu L. C. (2008). Distribution and seasonal dynamics of roots in a revegetated stand of *Artemisia ordosica* kracsh. in the tengger desert (North China). Arid. Land. Res. Manage. 22, 195–211. doi: 10.1080/15324980802182980

[B89] Zhang H. H. Xu N. Li X. Long J. H. Sui X. Wu Y. N.. (2018). Arbuscular mycorrhizal fungi (*Glomus mosseae*) improves growth, photosynthesis and protects photosystem II in leaves of *Lolium perenne* l. @ in cadmium contaminated soil. Front. Plant Sci. 9. doi: 10.3389/fpls.2018.01156 PMC6099091 30150997

[B90] Zhao S. Chen A. Chen C. Li C. Xia R. Wang X. (2019). Transcriptomic analysis reveals the possible roles of sugar metabolism and export for positive mycorrhizal growth responses in soybean. Physiol. Plant 166, 712–728. doi: 10.1111/ppl.12847 30288747

[B91] Zhu X. C. Song F. B. Liu S. Q. Liu T. D. (2011). Effects of arbuscular mycorrhizal fungus on photosynthesis and water status of maize under high temperature stress. Plant Soil 346, 189–199. doi: 10.1007/s11104-011-0809-8

[B92] Zou Y. N. Wang P. Liu C. Y. Ni Q. D. Zhang D. J. Wu Q. S. (2017). Mycorrhizal trifoliate orange has greater root adaptation of morphology and phytohormones in response to drought stress. Sci. Rep. 7, 41134. doi: 10.1038/srep41134 28106141 PMC5247675

[B93] Zou Y. N. Zhang F. Srivastava A. K. Wu Q. S. Kuca K. (2021). Arbuscular mycorrhizal fungi regulate polyamine homeostasis in roots of trifoliate orange for improved adaptation to soil moisture deficit stress. Front. Plant Sci. 11. doi: 10.3389/fpls.2020.600792 PMC7835339 33510746

