# Supplementary figures and images for: Effects of arbuscular mycorrhizal fungi on plant growth and herbivore infestation depend on availability of soil water and nutrients
Source: Front Plant Sci. 2023 Jan 26;14:1101932. doi: 10.3389/fpls.2023.1101932 (PMC9909235; doi:10.3389/fpls.2023.1101932)

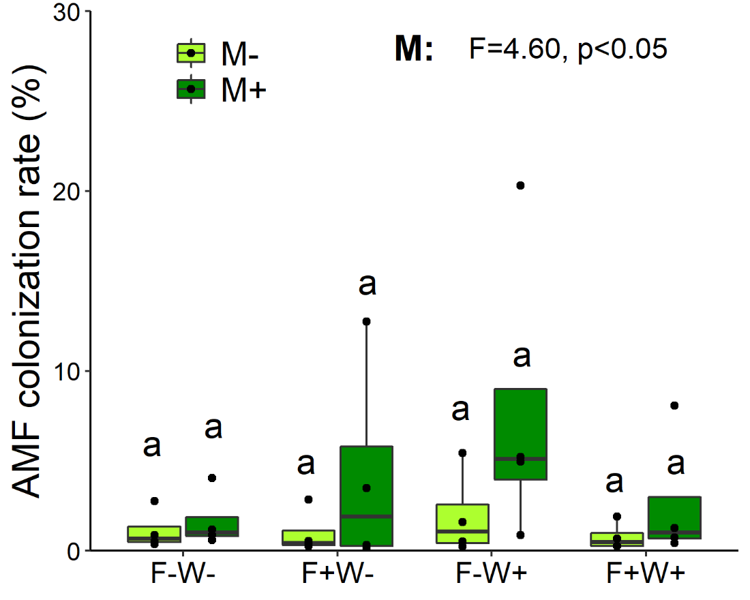

Supplement: Supplementary file 1 [file Image_1.tif]
